# Age-Related Association between Sex and Postoperative Atrial Fibrillation in Non-Cardiac Surgery Patients: Observational Cohort Study

**DOI:** 10.31083/j.rcm2409271

**Published:** 2023-09-25

**Authors:** Ah Ran Oh, Ji-Hye Kwon, Jungchan Park, Jong-Hwan Lee

**Affiliations:** ^1^Department of Anesthesiology and Pain Medicine, Samsung Medical Center, Sungkyunkwan University School of Medicine, 06351 Seoul, Republic of Korea

**Keywords:** cardiac event, atrial fibrillation, non-cardiac surgery

## Abstract

**Background:**

Postoperative atrial fibrillation (POAF) is a common complication that has shown conflicting results regarding sex differences. The potential effect of age on this association has not been adequately explored. We hypothesized that younger males would have a higher risk of POAF than females and that this difference would vary by age group.

**Methods:**

In this observational cohort study, we enrolled consecutive patients who underwent non-cardiac surgery between January 2011 and June 2019 at our institution and excluded those with preoperative atrial fibrillation and those undergoing sex-specific surgery. We stratified the patients into four groups based on their sex and age: females younger than 50 years, females older than 50 years, males younger than 50 years, and males older than 50 years. The primary outcome was the incidence of POAF.

**Results:**

Of the 141,337 patients included in the study, 6414 (4.5%) were treated for POAF. The incidence of POAF was highest in males older than 50 years (7.4%), followed by females older than 50 years (4.6%), males younger than 50 years (2.1%), and females younger than 50 years (1.9%). After adjusting for potential confounding factors, the risk of POAF was significantly increased in all groups compared with females younger than 50 years, with an odds ratio (OR) of 2.43 (95% confidence interval [CI]: 2.17–2.73, *p* < 0.001) for females older than 50 years, 1.19 (95% CI: 1.05–1.35, *p* = 0.01) for males younger than 50 years, and 4.39 (95% CI: 3.91–4.94, *p* < 0.001) for males older than 50 years. The OR for POAF risk according to sex peaked between 60 and 70 years old and decreased gradually thereafter.

**Conclusions:**

Our study suggests that sex and age are important factors associated with the risk of POAF in non-cardiac surgery patients and that sex-specific and age-specific risk stratification and interventions might be needed to prevent and manage POAF in non-cardiac surgery patients. Further studies are needed to better understand the underlying mechanisms of sex and age differences in POAF and to develop more targeted and effective interventions to reduce the incidence of this common postoperative complication.

## 1. Introduction

Postoperative atrial fibrillation (POAF) is characterized by new-onset atrial fibrillation in patients without a history of the condition, and is the most frequent postoperative complication [1]. POAF is associated with significant morbidity and mortality, including an increased risk of stroke and prolonged hospital stays [2]. Although the exact mechanism leading to POAF development has not been fully investigated, advanced age has long been considered one of the strongest predictors, with incidence rates sharply increasing after the age of 50 [3, 4]. This has commonly been explained by the normal aging process or the higher prevalence of comorbidities found in older populations, although changes in sex hormones have also been suggested as a contributing factor [5, 6, 7].

Sex is a significant variable in the development of POAF. However, previous studies have reported conflicting results. Although the most recent study reported no significant sex difference in the development of POAF after cardiac surgery [8], it is commonly known that POAF is more frequently found in male patients [9, 10, 11]. This difference is frequently attributed to the effect of estrogen, which can theoretically delay the onset of atrial fibrillation by regulating ion channels [5, 6]. However, the effects of sex hormones on POAF in real-world patients are complex. Testosterone has also shown protective effects against atrial fibrillation, with hormone replacement therapy decreasing atrial fibrillation [7, 12]. Moreover, estrogen is related to adverse outcomes in patients who develop atrial fibrillation, despite the protective effect against its development [13, 14]. Therefore, given different underlying mechanisms of POAF and the complexity of the effects of sex hormones, sex and age differences in POAF need to be investigated in real-world data. Our study hypothesis was that younger males would be at a higher risk of POAF than females and that the increase in risk would change with increasing age. To compare the risk of POAF, we stratified patients into four groups based on their sex and age: females younger and older than 50 years and males younger and older than 50 years. We chose 50 years as the age cut-off for stratification because it is commonly the age of menopause in females. Our findings could have important implications for preventing and managing POAF in non-cardiac surgery patients, particularly in light of an aging population and the increasing demand for non-cardiac surgery. A better understanding of age-related differences in the association between sex and POAF could enable the development of more targeted and effective interventions to reduce the incidence of this common postoperative complication.

## 2. Methods

### 2.1 Data Curation and Study Population 

We established the Samsung Medical Center Non-cardiac Operation registry (KCT 0006363), which comprises a de-identified cohort of 203,787 consecutive adult patients who underwent non-cardiac surgery with general or regional anesthesia at Samsung Medical Center, Seoul, Korea, between January 2011 and June 2019. This registry was created using the institutional electronic archive system, which holds the medical records of more than 4 million patients and contains more than 900 million laboratory findings and 200 million prescriptions. We obtained raw data from this system using Clinical Data Warehouse Darwin-C, an electronic tool for retrieving de-identified medical records. The mortality data in the records are consistently updated and confirmed with the National Population Registry of the Korean National Statistical Office using unique personal identification numbers for mortalities following hospital discharge.

An independent team of investigators used preoperative evaluation sheets to collect patients’ preoperative data: demographic information, medical history, and blood test results. Pre- and postoperative diagnoses were collected using International Classification of Diseases-10 codes from hospital progress notes, nursing charts, discharge notes, examination results, and drug prescriptions. To evaluate the effect of sex and age on POAF, which is defined as the new onset of atrial fibrillation after surgery, we excluded patients who had preexisting atrial fibrillation and those who underwent sex-specific procedures (i.e., breast, gynecologic, or urologic procedures) from the analysis.

We classified the patients into four groups according to their sex and age: females younger than 50 years, females older than 50 years, males younger than 50 years, and males older than 50 years. Furthermore, to gain more insight into the data, we created subgroups based on age in 10-year intervals. We also estimated the risk of POAF according to sex in the subgroups.

### 2.2 Definitions and Study Endpoints 

We categorized surgical procedures into risk strata in accordance with the European Society of Cardiology/European Society of Anesthesiology guidelines for non-cardiac surgery [1]. The preoperative Charlson Comorbidity Index was calculated based on the patients’ preoperative diagnoses using International Classification of Diseases-10 codes [15]. The primary outcome of interest was the occurrence of POAF requiring therapeutic intervention during a hospital stay within 30 days of non-cardiac surgery.

### 2.3 Statistical Analysis

Continuous variables are reported as the mean with standard deviation or median with interquartile range, and they were compared using appropriate statistical tests such as t-tests or Mann-Whitney tests. Categorical variables are reported as numbers and percentages, and they were compared using the chi-square test or Fisher’s exact test, as appropriate. To balance the distribution of covariates while maintaining the total number of patients, we used the inverse probability weighting (IPW) method to adjust for all available covariates. We used stabilized weights that were inversely proportional to the marginal probability of sex to balance the weights. We assessed the balance of covariates using the absolute standardized difference (ASD) and considered a difference of less than 10% to be negligible. To compare the risk of POAF, we used logistic regression models, and we report the results as odds ratio (OR) with 95% confidence intervals (CIs). All statistical analyses were performed using R version 4.2.0 (Vienna, Austria; http://www.R-project.org/).

## 3. Results

### 3.1 Baseline Characteristics

We excluded 1923 patients with preoperatively detected atrial fibrillation and 60,527 patients who underwent sex-specific procedures, resulting in a total of 141,337 patients available for analysis. The number of patients in each group was as follows: 24,712 (17.5%) females younger than 50 years, 43,744 (31.0%) females older than 50 years, 27,277 (19.3%) males younger than 50 years, and 45,604 (32.3%) males older than 50 years. Among all included patients, 6414 (4.5%) developed POAF. Table 1 shows the baseline characteristics and the incidence of POAF according to sex and age older or younger than 50 years. Older patients had significantly higher incidences of risk factors such as comorbidities and higher-risk procedures, whereas younger patients tended to show more habitual risk factors.

**Table 1.** **Baseline characteristics and outcomes according to sex and age of 50 years old**.

		Females younger than 50 years (N = 24,712)	Females older than 50 years (N = 43,744)	Males younger than 50 years (N = 27,277)	Males older than 50 years (N = 45,604)	*p*-value
Male		0 (0.0)	0 (0.0)	27,277 (100.0)	45,604 (100.0)	<0.001
Age		38.5 (±8.8)	64.0 (±8.8)	37.0 (±9.7)	63.6 (±8.3)	<0.001
Body mass index		22.9 (±3.8)	24.5 (±3.6)	25.0 (±3.7)	24.2 (±3.2)	<0.001
ASA physical classification						
	I	16,926 (68.5)	11,485 (26.3)	17,298 (63.4)	9514 (20.9)	<0.001
	II	7271 (29.4)	29,432 (67.3)	9137 (33.5)	30,915 (67.8)	<0.001
	III	460 (1.9)	2687 (6.1)	745 (2.7)	4849 (10.6)	<0.001
	IV	46 (0.2)	133 (0.3)	81 (0.3)	292 (0.6)	<0.001
	V	9 (0.0)	7 (0.0)	16 (0.1)	34 (0.1)	<0.001
Habitual risk factor						
	Current alcohol	4762 (19.3)	2628 (6.0)	11,308 (41.5)	12,263 (26.9)	<0.001
	Current smoking	586 (2.4)	461 (1.1)	6326 (23.2)	5500 (12.1)	<0.001
Previous disease						
	Hypertension	1273 (5.2)	17,479 (40.0)	2606 (9.6)	18,130 (39.8)	<0.001
	Diabetes	618 (2.5)	6573 (15.0)	1266 (4.6)	9709 (21.3)	<0.001
	Chronic kidney disease	119 (0.5)	693 (1.6)	230 (0.8)	1293 (2.8)	<0.001
	Dialysis	59 (0.2)	153 (0.3)	95 (0.3)	260 (0.6)	<0.001
	Stroke	137 (0.6)	1194 (2.7)	287 (1.1)	1786 (3.9)	<0.001
	Coronary artery disease	45 (0.2)	1201 (2.7)	104 (0.4)	1893 (4.2)	<0.001
	Heart failure	12 (0.0)	169 (0.4)	29 (0.1)	196 (0.4)	<0.001
	Arrhythmia	67 (0.3)	354 (0.8)	96 (0.4)	308 (0.7)	<0.001
	Peripheral artery disease	9 (0.0)	55 (0.1)	70 (0.3)	384 (0.8)	<0.001
	Aortic disease	12 (0.0)	112 (0.3)	31 (0.1)	452 (1.0)	<0.001
	Valvular heart disease	21 (0.1)	91 (0.2)	22 (0.1)	46 (0.1)	<0.001
	Chronic obstructive pulmonary disease	48 (0.2)	452 (1.0)	87 (0.3)	2183 (4.8)	<0.001
Preoperative blood laboratory tests						
	Hemoglobin, g/dL	12.7 (±1.3)	12.7 (±1.4)	14.9 (±1.6)	13.8 (±1.9)	<0.001
	Creatinine, mg/dL	0.7 (±0.5)	0.8 (±0.6)	1.0 (±0.8)	1.0 (±0.8)	<0.001
Operative variables						
	General anesthesia	22,786 (92.2)	34,497 (78.9)	24,256 (88.9)	41,197 (90.3)	<0.001
	Emergency operation	1499 (6.1)	2733 (6.2)	2111 (7.7)	3279 (7.2)	<0.001
	Operation duration, min	122.9 (±95.5)	125.2 (±90.3)	129.1 (±104.3)	154.8 (±108.1)	<0.001
	Intraoperative transfusion	740 (3.0)	1798 (4.1)	646 (2.4)	2002 (4.4)	<0.001
	Intraoperative inotropics infusion	1174 (4.8)	4198 (9.6)	1277 (4.7)	6841 (15.0)	<0.001
Surgical risk						
	Mild	12,124 (49.1)	16,851 (38.5)	13,574 (49.8)	10,005 (21.9)	<0.001
	Intermediate	11,436 (46.3)	24,258 (55.5)	11,898 (43.6)	29,049 (63.7)	<0.001
	High	1152 (4.7)	2635 (6.0)	1805 (6.6)	6550 (14.4)	<0.001
Surgery types						<0.001
	Neuroendocrine	5895 (23.9)	3693 (8.4)	2156 (7.9)	1281 (2.8)	
	Lung	996 (4.0)	4071 (9.3)	1034 (3.8)	5858 (12.8)	
	Head					
	Neck	6658 (26.9)	7794 (17.8)	8781 (32.2)	7598 (16.7)	
	Stomach	1615 (6.5)	3035 (6.9)	1783 (6.5)	6046 (13.3)	
	Hepatobiliary	2854 (11.5)	4478 (10.2)	3174 (11.6)	6545 (14.4)	
	Colorectal	1630 (6.6)	4272 (9.8)	1964 (7.2)	5874 (12.9)	
	Bone					
	Skin etc	5064 (20.5)	16,401 (37.5)	8385 (30.7)	12,402 (27.2)	
Outcomes						
	Postoperative atrial fibrillation	461 (1.9)	1992 (4.6)	581 (2.1)	3380 (7.4)	<0.001
	One-year mortality	375 (1.5)	1361 (3.1)	502 (1.8)	2810 (6.2)	<0.001
	Three-year mortality	823 (3.3)	2718 (6.2)	1022 (3.7)	5720 (12.5)	<0.001
	Acute kidney injury	213 (0.9)	846 (1.9)	493 (1.8)	2027 (4.4)	<0.001
	Stage 1	162 (0.7)	698 (1.6)	412 (1.5)	1684 (3.7)	<0.001
	Stage 2	24 (0.1)	89 (0.2)	46 (0.2)	212 (0.5)	<0.001
	Stage 3	27 (0.1)	59 (0.1)	35 (0.1)	131 (0.3)	<0.001

Data are presented as n (%) or mean (±standard deviation) or median (interquartile). Surgical risk was stratified according to 2014 European Society of Cardiology/European Society of Anaesthesiology guidelines. ASA, American Society of Anesthesiologists.

### 3.2 Risk of POAF According to Sex and Age

The highest incidence of POAF occurred in males older than 50 years (7.4%), followed by females older than 50 years (4.6%), males younger than 50 years (2.1%), and females younger than 50 years (1.9%). The risk of POAF in each group was compared with that of females younger than 50 years (Table 2). The unadjusted analysis revealed an odds ratio (OR) of 2.51 (95% CI: 2.27–2.78, *p* < 0.001) for females older than 50 years, 1.14 (95% CI: 1.01–2.30, *p* = 0.03) for males younger than 50 years, and 4.21 (95% CI: 3.82–4.65, *p* < 0.001) for males older than 50 years. We adjusted for all the covariates listed in Table 1. An ASD lower than 10% indicated successful variable balancing among the groups. After adjustment, the difference in POAF risk persisted. Compared with females younger than 50 years, the adjusted OR was 2.43 (95% CI: 2.17–2.73, *p* < 0.001) for females older than 50 years, 1.19 (95% CI: 1.05–1.35, *p* = 0.01) for males younger than 50 years, and 4.39 (95% CI: 3.91–4.94, *p* < 0.001) for males older than 50 years.

**Table 2.** **Risk of postoperative atrial fibrillation according to sex and age of 50 years old**.

	Females younger than 50 years (N = 24,712)	Females older than 50 years (N = 43,744)	Males younger than 50 years (N = 27,277)	Males older than 50 years (N = 45,604)
Number and incidence	461 (1.9)	1992 (4.6)	581 (2.1)	3380 (7.4)
Unadjusted OR (95% CI)	1	2.51 (2.27–2.78)	1.14 (1.01–2.30)	4.21 (3.82–4.65)
*p*-value		<0.001	0.03	<0.001
IPW adjusted OR (95% CI)	1	2.43 (2.17–2.73)	1.19 (1.05–1.35)	4.39 (3.91–4.94)
*p*-value		<0.001	0.01	<0.001

Data are presented as n (%) All variables in Table 1 were adjusted for. OR, odds ratio; CI, confidence interval; IPW, inverse probability weighting.

### 3.3 Risk of POAF According to Sex in Subgroups of Ten-Year Age Intervals

Patients were stratified into age intervals of ten years. The number of patients in each subgroup was 11,431 (8.1%) younger than 30 years, 14,479 (10.2%) between 30 and 40 years, 226,865 (16.2%) between 40 and 50 years, 36,224 (25.4%) between 50 and 60 years, 32,504 (23.0%) between 60 and 70 years, 19,946 (14.1%) between 70 and 80 years, and 3868 (2.7%) older than 80 years (Table 3). The risk of POAF did not differ according to sex in patients younger than 40 years, but the risk of POAF was significantly higher in male patients older than 40 years. The adjusted analysis showed that the risk difference, shown as OR, was 1.28 (95% CI: 1.07–1.54, *p* = 0.01) in the group between 40 and 50 years and increased with age, peaking at 1.89 (95% CI: 1.72–2.08, *p* < 0.001) in patients between 60 and 70 years old. In patients older than 70 years, the OR decreased.

**Table 3.** **Association between postoperative atrial fibrillation and sex according age groups**.

		Female	Male	Unadjusted OR (95% CI)	*p*-value	IPW adjusted OR (95% CI)	*p*-value
Age under 30		N = 4584	N = 6847				
	Postoperative atrial fibrillation	94 (2.1)	122 (1.8)	0.87 (0.66–1.14)	0.3	0.91 (0.69–1.21)	0.51
Age between 30–40		N = 7068	N = 7411				
	Postoperative atrial fibrillation	125 (1.8)	140 (1.9)	1.07 (0.84–1.37)	0.59	1.12 (0.88–1.43)	0.37
Age between 40–50		N = 11,450	N = 11,415				
	Postoperative atrial fibrillation	218 (1.9)	272 (2.4)	1.26 (1.05–1.51)	0.01	1.28 (1.07–1.54)	0.01
Age between 50–60		N = 17,839	N = 18,405				
	Postoperative atrial fibrillation	478 (2.7)	805 (4.4)	1.66 (1.48–1.86)	<0.001	1.65 (1.47–1.85)	<0.001
Age between 60–70		N = 15,284	N = 17,220				
	Postoperative atrial fibrillation	649 (4.2)	1331 (7.7)	1.89 (1.72–2.08)	<0.001	1.89 (1.72–2.08)	<0.001
Age between 70–80		N = 9962	N = 9984				
	Postoperative atrial fibrillation	679 (6.8)	1091 (10.9)	1.68 (1.52–1.85)	<0.001	1.65 (1.50–1.83)	<0.001
Age over 80		N = 2269	N = 1599				
	Postoperative atrial fibrillation	210 (9.3)	200 (12.5)	1.40 (1.14–1.72)	0.001	1.38 (1.11–1.70)	0.003

Data are presented as n (%). OR, odds ratio; CI, confidence interval; IPW, inverse probability weighting.

## 4. Discussion

In this large retrospective study of more than 140,000 patients who underwent non-cardiac surgery, we found that the incidence of POAF varied according to sex and age. Male patients older than 50 years had the highest risk of developing POAF, followed by female patients older than 50 years, male patients younger than 50 years, and female patients younger than 50 years. We also found that sex differences in the risk of POAF varied with age: whereas there was no significant difference in POAF risk between the sexes in patients younger than 40 years, male patients had a significantly higher risk of POAF than female patients in the older age groups. The POAF risk difference according to sex increased with age and peaked in patients between 60 and 70 years old. These findings could have meaningful clinical implications for the risk stratification and management of patients undergoing non-cardiac surgery, and they highlight the need for sex- and age-specific risk assessment for POAF.

Understanding epidemiological differences in the incidence and risk factors of POAF is crucial for improving patient outcomes and reducing healthcare costs. POAF is associated with increased morbidity, cardiovascular disease–related mortality, and length of hospital stay, which can result in increased healthcare costs and decreased patient quality of life [2]. Identifying patients who are at increased risk of developing POAF can allow targeted interventions and monitoring to prevent or manage the condition [16]. Furthermore, understanding differences in the risk factors for POAF between different patient groups can inform personalized treatment and prevention strategies [17]. Previous studies have reported differences in the incidence of POAF among various patient populations, including differences by age, sex, comorbidities, and surgical procedures [18].

Although advanced age is consistently recognized as a major risk factor for atrial fibrillation, the effect of sex on this risk has remained uncertain, with inconsistent findings reported in previous studies [19]. Various aspects of sex differences in atrial fibrillation have been investigated, including prevalence, clinical presentation, associated comorbidities, and treatment outcomes [20, 21]. Whereas the prevalence of age-related atrial fibrillation is higher in males [22], females often experience worse and atypical symptoms, lower quality of life, and a higher risk of adverse events such as stroke, possibly due to the presence of estrogen [13, 14]. Additionally, females are more susceptible to atrial fibrillation recurrences than males [14, 20]. It is worth noting that the underlying mechanism of POAF might differ from that of atrial fibrillation, with perioperative factors such as surgical stress, inflammation, and anesthesia use potentially playing a significant role [1, 23]. The objective of our study was to investigate the risk of POAF by sex and age. Our primary analysis revealed that older male patients had the highest incidence of POAF, and young females had a significantly lower risk than older females and young males.

The primary result of our study is consistent with previous findings about the effects of sex on the development of atrial fibrillation [22]. It is most frequently explained by citing the protective effect of estrogen on the heart through the regulation of ion channels and anti-inflammatory effects [5, 6]. However, our further analysis evaluating sex-differences in the risk of POAF in different age groups stratified by ten-year intervals revealed that the risk of POAF did not differ significantly according to sex in patients younger than 40 years. This finding cannot be fully explained by the protective effect of estrogen against the development of atrial fibrillation, especially because the risk difference, measured as an OR, increased until 70 years of age, despite the significant decline in estrogen levels after menopause [24]. This discrepancy could be related to the effects of testosterone in male patients [25, 26]; the landmark cohort of the Framingham Heart Study showed an association between low testosterone levels and an increased risk of atrial fibrillation in males older than 80 years, which is consistent with our findings [7]. Additionally, hormone replacement therapy has been shown to decrease the incidence of atrial fibrillation [12]. Interestingly, low testosterone levels have also shown a protective effect against atrial fibrillation in females [26].

Another possible explanation for the absence of a sex difference in POAF risk in patients younger than 40 years is related to the poorer prognosis of female patients who have already developed atrial fibrillation [13, 14]. In addition, there is a well-documented decline in estrogen levels that begins around age 40, well before menopause [27]. The subgroups that demonstrated a sex-difference in POAF were those in which estrogen levels had declined in females while testosterone levels remained high in males. In this scenario, the development of POAF seems to be consistent with the prognosis of atrial fibrillation, which is worsened by estrogen, rather than the development of atrial fibrillation, in which estrogen has protective effects [13, 14]. However, the overall association between atrial fibrillation and sex hormones appears to be complex and requires further investigation.

Our study has several limitations that need to be acknowledged. First, the retrospective design of the study does not allow us to establish causality. Secondly, we had no control over perioperative care during the lengthy study period. Although our institution had a protocol based on current guidelines, clinical decisions were often at the discretion of the attending physicians. Furthermore, since most patients did not have continuous monitoring, transient and self-limiting episodes of POAF may have gone undetected, and we could only include cases that were actually treated. Thirdly, our findings cannot be extrapolated to other patient populations because this study was conducted at a single center and did not consider ethnic differences. Fourthly, not all patients in our sample had detailed preoperative cardiac evaluations such as left ventricular ejection fraction or coronary artery angiograms, which might have influenced our results. Therefore, there is a possibility that other known risk factors for POAF, which were not accounted for in our study, might have influenced our findings. Additionally, we were unable to evaluate the effect of perioperative cardiac medical treatment, as relevant data was not fully available. Fifth, we are unable to propose any modality to minimize POAF, such as sex hormone replacement therapy, so that requires further investigation. Despite these limitations, our study has demonstrated age-related sex differences in the development of POAF in real-world data.

## 5. Conclusions

Our study suggests that sex and age are important factors associated with the risk of POAF in non-cardiac surgery patients and that sex-specific and age-specific risk stratification and interventions might be needed to prevent and manage POAF in non-cardiac surgery patients. Further studies are needed to better understand the underlying mechanisms of sex and age differences in POAF and to develop more targeted and effective interventions to reduce the incidence of this common postoperative complication.

## Data Availability

The datasets used and/or analyzed during the current study are available from the corresponding author on reasonable request.
